# Genome-Wide Transcriptome and Proteome Analysis on Different Developmental Stages of *Cordyceps militaris*

**DOI:** 10.1371/journal.pone.0051853

**Published:** 2012-12-14

**Authors:** Yalin Yin, Guojun Yu, Yijie Chen, Shuai Jiang, Man Wang, Yanxia Jin, Xianqing Lan, Yi Liang, Hui Sun

**Affiliations:** 1 State Key Laboratory of Virology, College of Life Sciences, Wuhan University, Wuhan, Hubei Province, People’s Republic of China; 2 Department of Clinical Immunology, Guangdong Medical College, Dongguan, People’s Republic of China; 3 Key Laboratory of Fermentation Engineering (Ministry of Education), Hubei University of Technology, Wuhan, People’s Republic of China; 4 Key Laboratory of Combinatorial Biosynthesis and Drug Discovery (Wuhan University), Ministry of Education, Wuhan, People’s Republic of China; University of Georgia, United States of America

## Abstract

**Background:**

*Cordyceps militaris*, an ascomycete caterpillar fungus, has been used as a traditional Chinese medicine for many years owing to its anticancer and immunomodulatory activities. Currently, artificial culturing of this beneficial fungus has been widely used and can meet the market, but systematic molecular studies on the developmental stages of cultured *C. militaris* at transcriptional and translational levels have not been determined.

**Methodology/Principal Findings:**

We utilized high-throughput Illumina sequencing to obtain the transcriptomes of *C. militaris* mycelium and fruiting body. All clean reads were mapped to *C. militaris* genome and most of the reads showed perfect coverage. Alternative splicing and novel transcripts were predicted to enrich the database. Gene expression analysis revealed that 2,113 genes were up-regulated in mycelium and 599 in fruiting body. Gene Ontology (GO) and Kyoto Encyclopedia of Genes and Genomes (KEGG) analysis were performed to analyze the genes with expression differences. Moreover, the putative cordycepin metabolism difference between different developmental stages was studied. In addition, the proteome data of mycelium and fruiting body were obtained by one-dimensional gel electrophoresis (1-DGE) coupled with nano-electrospray ionization liquid chromatography tandem mass spectrometry (nESI-LC-MS/MS). 359 and 214 proteins were detected from mycelium and fruiting body respectively. GO, KEGG and Cluster of Orthologous Groups (COG) analysis were further conducted to better understand their difference. We analyzed the amounts of some noteworthy proteins in these two samples including lectin, superoxide dismutase, glycoside hydrolase and proteins involved in cordycepin metabolism, providing important information for further protein studies.

**Conclusions/Significance:**

The results reveal the difference in gene expression between the mycelium and fruiting body of artificially cultivated *C. militaris* by transcriptome and proteome analysis. Our study provides an effective resource for the further developmental and medicinal research of this promising fungus.

## Introduction

Mushrooms, also known as macrofungi, have received considerable attention due to its rich set of biologically active compounds [1], [2]. *Cordyceps militaris*, an entomopathogenic fungus in Ascomycetes, is a popular traditional Chinese medicinal mushroom. It has been widely used as an herbal drug and a tonic food in East Asia and also being studied in the West recently because of its various biological activities such as antitumor, antioxidation and immunomodulatory effects [3]–[8]. Series of pharmacologically active ingredients, such as cordycepin, cordycepic acids, polysaccharide and cyclic peptides, have been found in *C. militaris* [9]–[15]. Moreover, a lectin purified from *C. militaris* exhibits mitogenic activity against mouse splenocytes [16] and a protein exerts effective antifungal and anticancer activities. Recently, a new antifungal peptide and a novel fibrinolytic enzyme have been identified from *C. militaris* [17]. Among these compounds, cordycepin arises most of the interests and is now undergoing Phase II antitumor clinical trials [18]. It has been found to display antibacterial, antifungal, antitumor, anti-inflammatory and anti-metastatic effects [19]–[24], but very few studies focus on the cordycepin production pathway.

The natural *C. militaris* is rare and costly, thus cannot meet the market needs. Fortunately, artificial culturing of *C. militaris* and its products are now available [25], [26]. It has been proved that the active components of cultured *C. militaris* are similar as the natural ones, which enables the substitution of wide *Cordyceps* by artificial ones [27]–[29]. Previously, gene expression patterns of different developmental stages of *C. militaris* were studied by expressed sequence tag (EST) technique and dissimilarities were found in the transcriptional profiles of fruiting body produced on rice medium and silkworm pupae [26]. Recently, the genome of *C. militaris* and the transcriptome of its mycelium on Sabouraud dextrose broth (SDB) medium and fruiting body on silkworm pupae have been sequenced and analyzed [30]. Thus, the transcriptome analysis of *C. militaris* cultivated on different medium is needed, which will facilitate the developmental and medicinal research of this beneficial fungus.

High-throughput RNA-seq using next-generation Illumina sequencing technique provides an effective and comprehensive method to deeply study the transcriptome of an organism such as human [31], yeast [32], rice [33], oriental fruit fly [34] and *Tetrahymena thermophila* [35]. With the development of sequencing technique and mycology, the genomes or transcriptomes of fungi have been increasingly obtained for further research, including *Metarhizium* species [36], *Coprinus cinerea* [37], *Trichoderma reesei* [38], *Schizophyllum commune* [39], *Laccaria bicolor* [40], [41], *Tuber melanosporum* [42], [43], *Postis placenta* [44], [45]. By mapping the sequenced transcriptome reads to a reference genome, the structure annotation can be improved and novel transcripts and alternative splicing can be predicted, which expand the current genome databases [31], [42], [46].

Proteomic one-dimensional or two-dimensional gel electrophoresis (1-DGE or 2-DGE) approaches combined with mass spectrometry (MS) have been widely used to identify interested proteins in fungi and many other organisms [47]–[52]. Since the expression of some genes is regulated at the transcriptional level while some other genes at the translational level [53], the combination of transcriptome and proteome analysis is required and has been widely applied [54]–[56].

In this study, we conducted the transcriptome and proteome analysis of different developmental stages of *C. militaris* (mycelium cultured on potato dextrose agar (PDA) and fruiting body cultured on rice medium). About 68 million reads were obtained from each sample and were mapped to the *C. militaris* genome. We found 2712 differentially expressed genes between mycelium and fruiting body. Functional annotations were used to investigate their difference including the cordycepin metabolism difference. In addition, 359 and 214 non-redundant proteins were identified from mycelium and fruiting body respectively by nESI-LC-MS/MS. Proteome analysis revealed the developmental-associated protein expression patterns including some noteworthy proteins. Our transcriptome and proteome analysis data enrich the current database of *C. militaris* and will facilitate the future developmental study and medicinal research on this famous fungus.

**Table 1:** Mapping results of *C. militaris* transcriptome.

Summary		Mycelium	Fruiting body
Clean reads		6,767,008	6,855,896
Mapping to genome	Total mapped reads	6,041,335 (89.28%)	5,710,126 (83.29%)
	Perfect match	4,775,276 (70.57%)	4,51,9187 (65.92%)
	≤5 bp mismatch	1,266,059 (18.71%)	1,190,939 (17.37%)
	Unique match	6,037,886 (89.23%)	5,718,977 (83.42%)
	Multi-position match	3,449 (0.05%)	682 (0.01%)
Mapping to gene	Total mapped reads	3,554,477 (52.53%)	3,049,818 (44.48%)
	Perfect match	2,844,130 (42.03%)	2,403,987 (35.06%)
	≤2 bp mismatch	710,347 (10.50%)	645,831 (9.42%)
	Unique match	3,554,316 (52.52%)	3049,611 (44.48%)
	Multi-position match	161 (0.00%)	207 (0.00%)

## Materials and Methods

### Sample Preparation

The mycelium inoculum from the active potato dextrose agar (PDA) slant of *C. militaris* was inoculated to PDA plate (diameter = 9 cm) and incubated at 22°C for 18 days in the dark, the fresh mycelium were collected for RNA isolation. On the other hand, the mycelium inoculum from the PDA slant was inoculated to rice medium in a canned bottles, then incubated at 22°C for 14 days in the dark and then for 6 days with a 16∶8 h dark/light cycle to form stromata, at last maintain 22°C for 30 days with the same condition to form mature fruiting body [26]. The fresh fruiting body was haversted for RNA isolation.

### RNA Isolation

Total-RNAs were extracted from mycelium and fruiting body of *C. militaris* respectively with Trizol reagent (Invitrogen, USA) according to the instructions of the manufacturer. RNA quality and concentration were evaluated by Agilent 2100 Bioanalyzer (Agilent Technologies, Santa Clara, CA).

**Figure 1:**
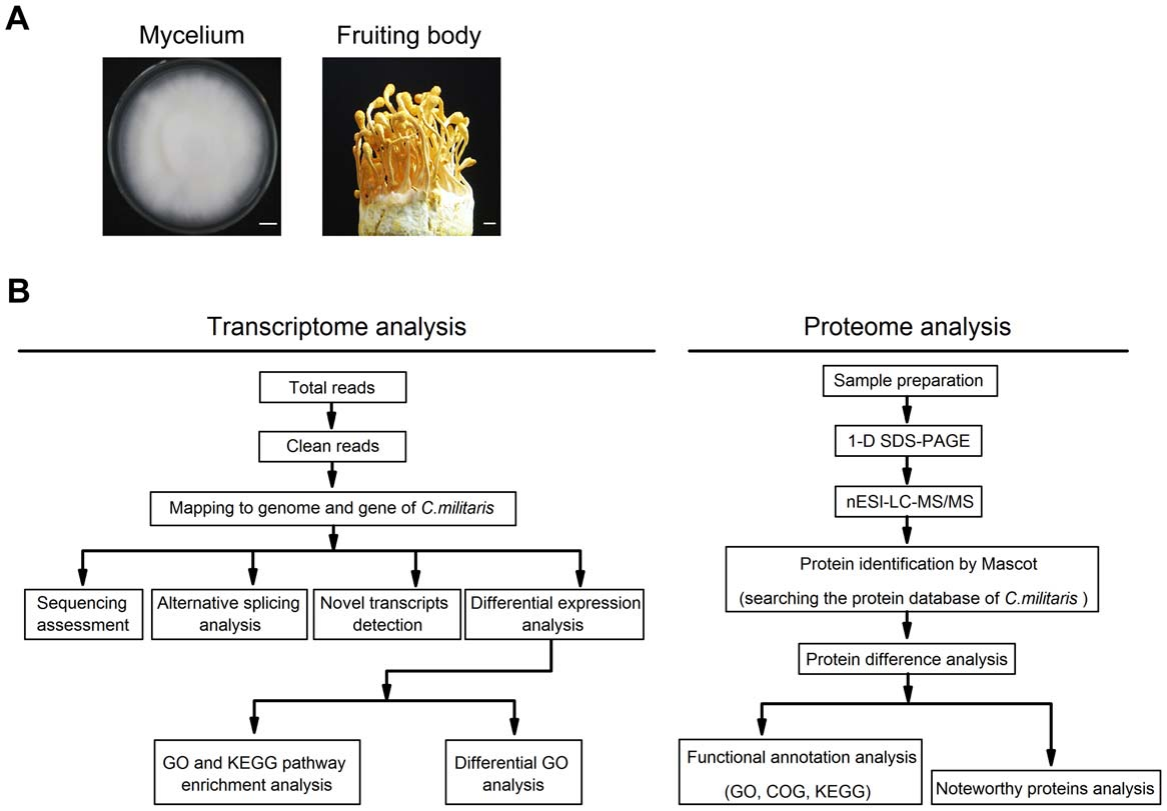
Pipelines of transcriptome and proteome analysis of *C. militaris*. (A) Mycelium and mature fruiting body images of *C. militaris*; scale bars, 1 cm. (B) Analysis Pipelines of transcriptome and proteome of *C. militaris*.

**Figure 2:**
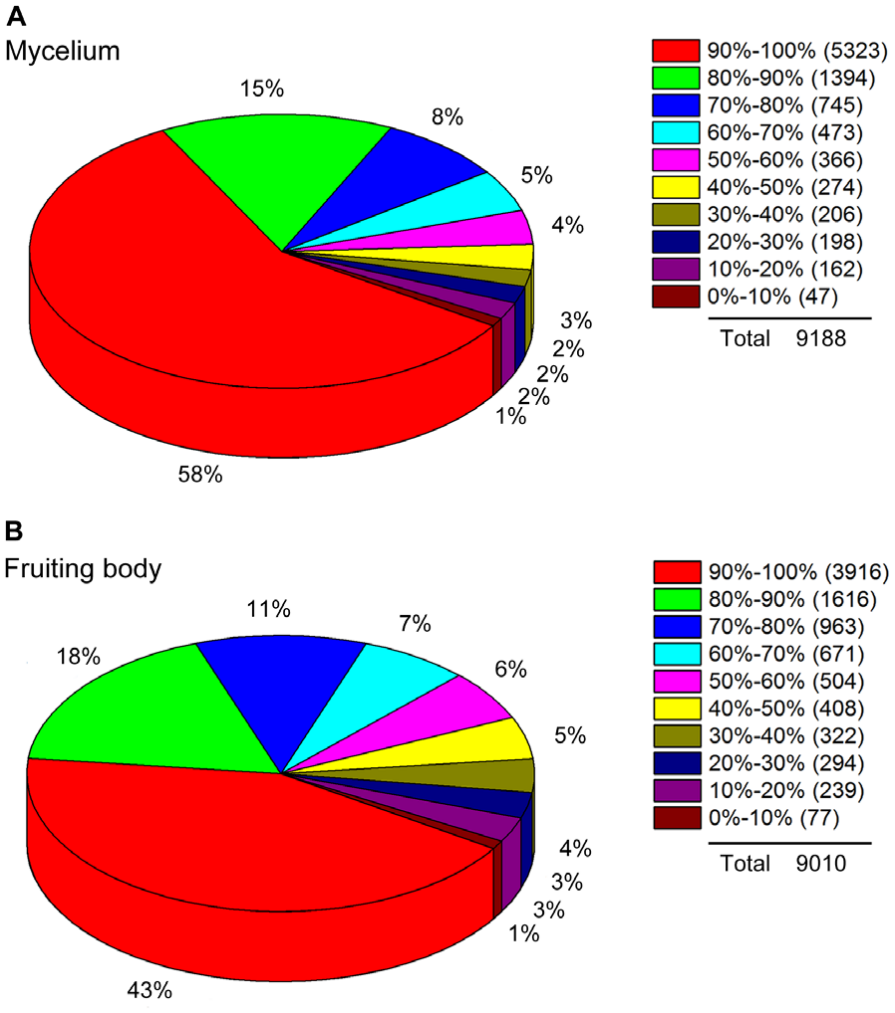
Distribution statistics of genes’ coverage. The pie charts showed the distributions of genes’ coverage of mycelium (A) and fruiting body (B) respectively. Gene coverage is the percentage of a gene covered by reads. The value equals to ratio of the number of bases in a gene covered by unique mapping reads to number of total bases in that gene.

### cDNA Library Preparation and Sequencing

A total of 20 µg RNA was equally isolated from the two samples for cDNA library construction. Beads with oligo (dT) were used to enrich poly(A) mRNA, then fragmentation buffer was added for interrupting mRNA to short fragment (200–700 nt) which were used as templates to synthesize the first-strand cDNA using random hexamer primer, the second-strand cDNA was synthesized by adding buffer, dNTPs, RNaseH and DNA polymerase I, respectively. The products were purified with QiaQuick PCR extraction kit and resolved with EB buffer for end reparation and adding poly (A). After that, the cDNA fragments were connected with sequencing adaptors and gel-electrophoresis was used to select cDNA fragments of 200 bp (±25 bp) in size as the templates for amplification with PCR. Two constructed cDNA library were sequenced using Illumina Hiseq™ 2000 at Beijing Genome Institute (BGI, Shenzhen, China).

### Data Processing

Raw images generated by sequencers were converted by Illumina GA Pipeline 1.6 to nucleotide sequences, which called raw reads. Clean reads were obtained by removing adaptor reads and low quality reads (Q ≤5), on which all following analysis are based. The clean reads were mapped to the genome and gene sequences of *C. militaris* [30] respectively using the Short Oligonucleotide Analysis Package SOAPaligner/soap2 [57], up to five bases mismatches were allowed in the genome alignment while up to two bases mismatches were allowed in gene alignment. The mapping results for two samples were shown in Table 1.

**Figure 3:**
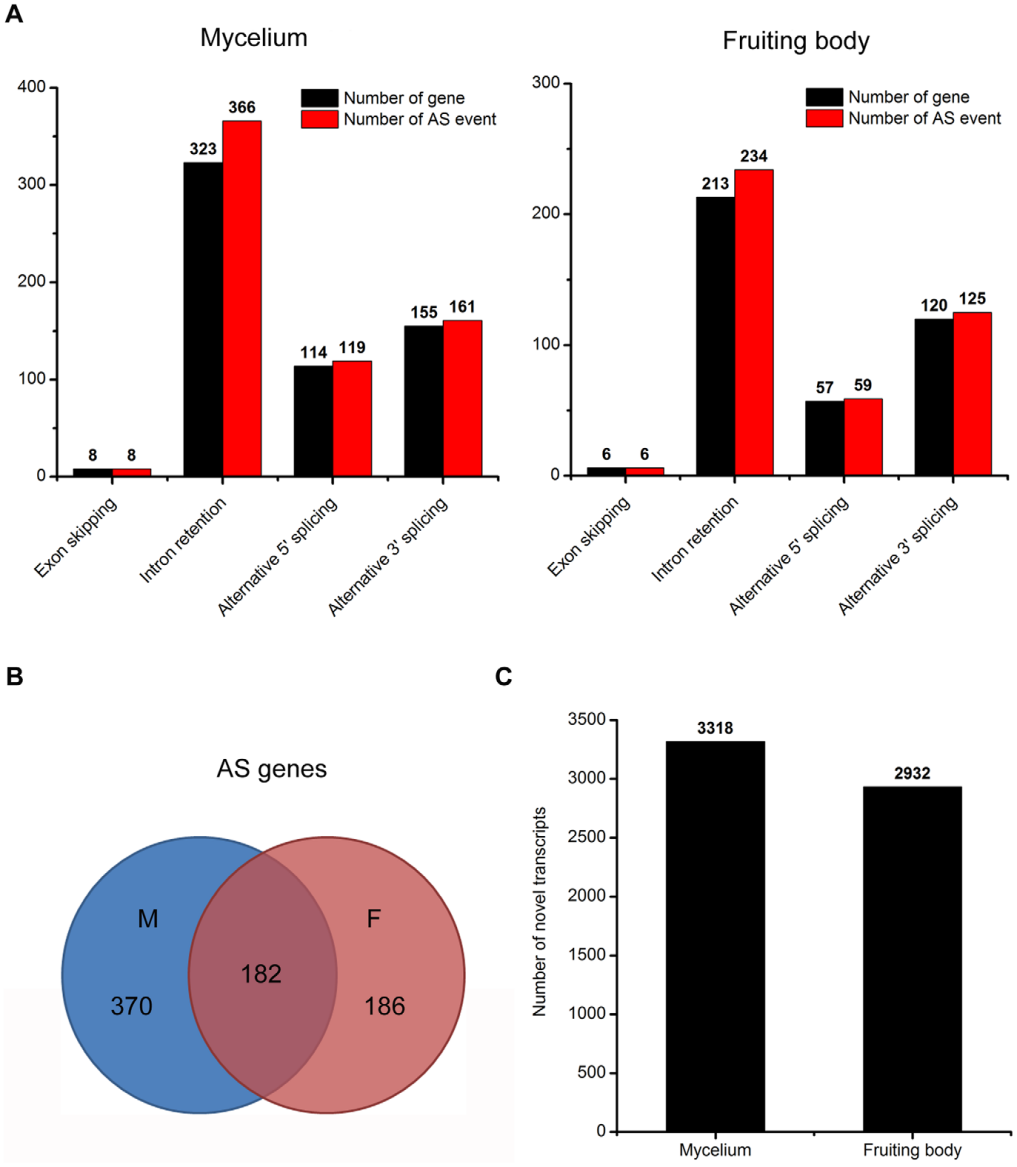
Alternative splicing (AS) and novel transcripts prediction. (A) Numbers of alternative splicing events and involved genes in different developmental stages of *C. militaris*. The x-axis represents types of alternative splicing events. (B) Prediction of novel transcripts in mycelium and fruiting body.

### Alternative Splicing and Novel Transcripts Predication

Alternative splicing events were classified into four basic types: exon skipping, intron retention, alternative 5′ splice site, alternative 3′ splice site. TopHat [58] (with all default parameters) was used to detect the splice junction sites which give information about boundaries and combinations of different exons in a transcript, Then all splice junction sites of the same gene are used to distinguish type of its alternative splicing event.

For novel transcripts prediction, the potential gene models found in intergenic regions (200 bp away from upstream or downstream genes) with lengths >150 bp and average coverage >2 were thought to be candidate of novel transcripts.

**Figure 4:**
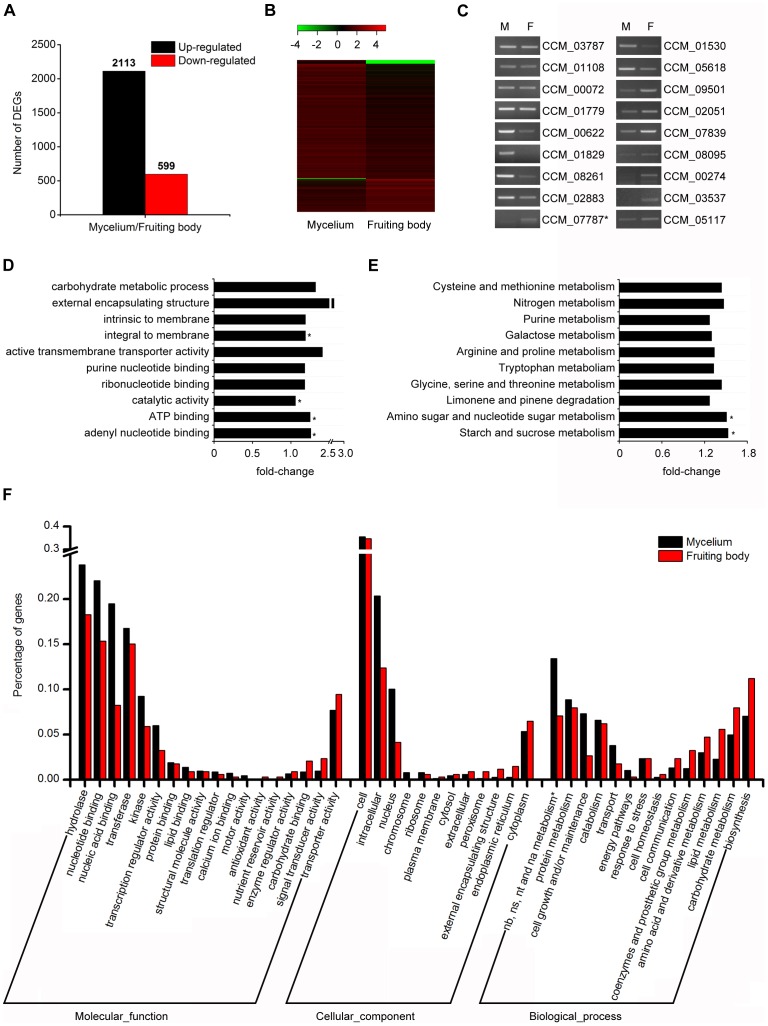
Comprehensive analysis of differentially expressed genes (DEGs) between mycelium and fruiting body of *C. militaris*. (A) Estimation of significantly up-regulated and down-regulated genes between different developmental stages. (B) The heat map of DEGs. log (base 10) value of RPKM data was used to draft the map. As is shown in the above brand, −4 value indicates the absence of particular genes in its corresponding transcriptome. The green color represents relatively lower expression and red color represents higher. (C) Verification of the gene expression by semi-quantitative RT-PCR. M: mycelium; F: fruiting body. The *actin* gene CCM_03787 was used as a reference. The detailed information of 17 selected genes was listed in Table 2. * The experimental result is not in accordance with the transcriptome data. (D) GO terms enrichment analysis. Ten terms that show smallest p value in three main categories (biological process, cellular component, molecular function) were chosen. * p≤0.05, it indicates the significant enriched GO terms in DEGs. (E) KEGG pathway enrichment analysis. Ten pathways that show smallest Q value were selected. * Q ≤0.05, it represents the significant enriched pathways in DEGs. X-axes of (D) and (E) represent the fold changes of GO terms (D) and KEGG pathways (E) of DEGs in comparison with the genome. (F) GO terms of DEGs. Crossbands in black represent up-regulated genes in mycelium compared with fruiting body, and crossbands in red represent up-regulated genes in fruiting body compared with mycelium. * represents nucleobase, nucleoside, nucleotide and nucleic acid metabolism. 1365 up-regulated genes in mycelium and 339 in fruiting body got GO annotation.

**Table 2:** The expression information in different stages of selected genes for semi-quantitative RT-PCR.

	Gene	Length	M_RPKM	F_RPKM	log2(M_RPKM/F_RPKM)	Annotation[Cordyceps militaris CM01]
Reference	CCM_03787	1128	1377.06	1039.26	0.41	actin
No_DEGs	CCM_01180	2892	115.48	67.58	0.77	Zinc finger domain-containing protein, GATA-type
	CCM_00072	1512	141.23	162.87	−0.20	Cutinase palindrome-binding protein
	CCM_01779	1065	100.39	93.29	0.10	vesicular integral-membrane protein VIP36 precursor
M_up	CCM_00622	1857	178.78	23.13	2.95	5′-nucleotidase
	CCM_01829	3006	66.64	6.33	3.40	ABC transporter, putative
	CCM_08261	12459	45.82	7.47	2.62	polyketide synthase, putative
	CCM_02883	3048	1298.19	210.54	2.62	beta-galactosidase
	CCM_07787*	591	69.50	0.00	16.08	Mannose-binding lectin
	CCM_01530	1617	1956.04	69.96	4.81	cytochrome P450 oxidoreductase OrdA-like protein
	CCM_05618	2718	138.71	5.43	4.68	C6 transcription factor
F_up	CCM_09501	762	150.64	2291.93	−3.93	O-methyltransferase family 3
	CCM_02051	3366	2.93	23.48	−3.00	phosphoribosyl transferase
	CCM_07839	2100	513.93	1853.32	−1.85	heat shock protein 90
	CCM_08095	1248	27.73	744.37	−4.75	chitin deacetylase
	CCM_00274	1986	2.41	57.79	−4.58	pantothenate transporter liz1
	CCM_03537	318	22.12	108491.00	−12.26	Hydrophobin 2
	CCM_05117	1332	582.34	1921.92	−1.72	1,3-beta-glucanosyltransferase Gel1

* The RT-PCR result was not in accordance with the *C. militaris* transcriptome data.

### Functional Annotation

The genomic data of *C. militaris* was obtained from NCBI (BioProject acession no. PRJNA41129). Functional annotation by gene ontology terms (GO; http://www.geneontology.org) [59] was downloaded from Uniprot (http://www.uniprot.org/uniprot) database. KEGG pathway and COG annotation was performed using Blastall software against Kyoto Encyclopedia of Genes and Genomes database [60] and Cluster of Orthologous Groups database [61] respectively with a cut-off *E* value of 1e−5.

### Differential Expression Analysis

RPKM method (Reads Per Kb per Million reads) [62] was used to calculate gene expression level. FDR (False Discovery Rate) [63] was developed as a criteria to identify the differential expression genes (DEGs) between two samples. In our analysis, the FDR ≤0.001 and the |log_2_RPKM ratio| ≥1 were set as the threshold to determine the significance of gene expression difference between mycelium and fruiting body stage of *C. militaris*. For function enrichment analysis, GO terms of DEGs were compared to the genome background and the corrected p value (p-value gone through Bonferroni Correction) less than 0.05 was set to judge the significantly enriched GO terms. The pathway enrichment anlysis was performed similarly using the KEGG database and the Q value (FDR analogue of the p-value) less than 0.05 was the threshold.

**Figure 5:**
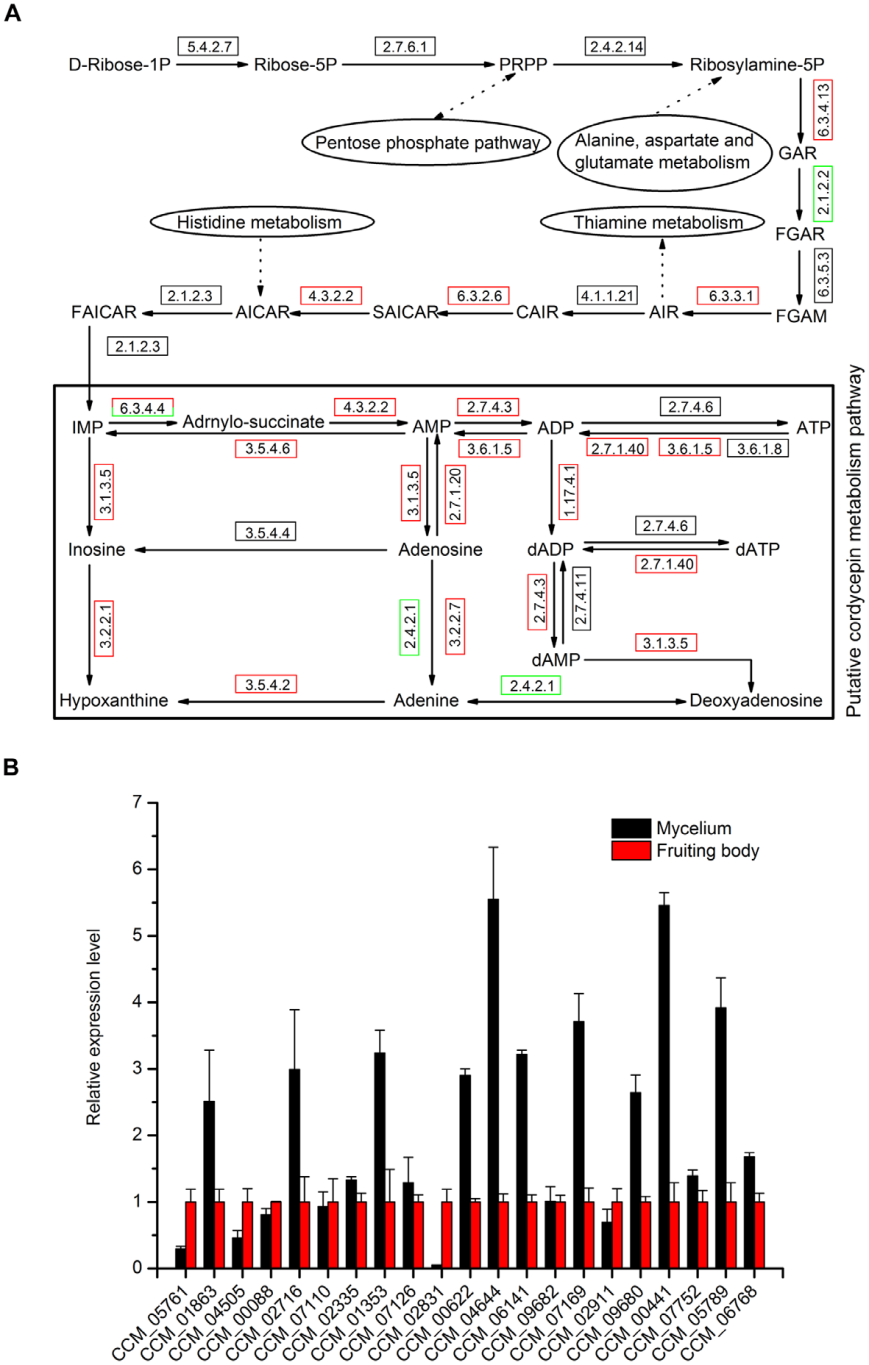
Differentially expressed genes associated with putative cordycepin metabolic pathway at different developmental stages. (A) Putative cordycepin metabolic pathway derived from purine metabolic pathway. Red boxes represent some genes in the steps are up-regulated in mycelium, while green boxes represent that in fruiting body. Black boxes mean the common expression level of genes in the steps between mycelium and fruiting body. The detailed information of genes involved in the pathway was listed in Table 3. (B) Validation of the gene expression in cordycepin metabolic pathway by quantitative RT-PCR. *Actin* gene CCM_03787 was the reference. The expression levels of randomly selected genes in fruiting body were set as control samples and those in mycelium were normalized to the control. The values are the results of three technical repetitions (mean ± SD).

**Table 3:** Differential expression of genes putatively involved in cordycepin metabolism pathway.

EC No.	Enzymes	Gene	M_RPKM	F_RPKM	log2(M_RPKM/F_RPKM)	Up/down in mycelium
1.17.4.1	Ribonucleotide reductase	CCM_05761	169.9	79.47	1.1	Up
		CCM_01863	51.61	24.4	1.05	Up
2.4.2.1	Purine nucleoside phosphorylase	CCM_04505	102.64	216.47	−1.08	Down
2.4.2.7	Adenine phosphoribosyltransferase	CCM_00088	190.6	199.88	−0.07	–
2.7.1.20	Adenosine kinase	CCM_02716	180.89	77.84	1.22	Up
2.7.1.40	pyruvate kinase	CCM_05734	44.72	4.47	3.32	Up
		CCM_07110	63.32	69.55	−0.14	–
2.7.4.3	Adenylate kinase	CCM_07479	90.15	47.03	0.94	–
		CCM_02335	18.39	18.57	−0.01	–
		CCM_01353	371.71	95.21	1.97	Up
		CCM_04983	22.4	36.3	−0.7	–
2.7.4.6	nucleoside-diphosphate kinase	CCM_07126	645.63	582.95	0.15	–
2.7.4.11	deoxyadenylate kinase	CCM_02335	18.39	18.57	−0.01	–
3.1.3.5	5′-nucleotidase	CCM_02831	34.78	61.89	−0.83	–
		CCM_00622	178.78	23.13	2.95	Up
		CCM_04644	43.38	12.27	1.82	Up
3.2.2.1	Purine nucleosidase	CCM_06141	55.97	15.93	1.81	Up
3.2.2.7	Adenosine nucleosidase	CCM_09682	8.62	7.72	0.16	–
3.5.4.2	Adenine deaminase	CCM_07169	11.35	2.94	1.95	Up
3.5.4.4	Adenosine deaminase	CCM_02911	7.99	9.31	−0.22	–
		CCM_09449	76.51	40.27	0.93	–
3.5.4.6	AMP deaminase	CCM_09680	272.77	98.58	1.47	Up
3.6.1.5	Adenosine diphosphatase	CCM_00441	75.19	31.6	1.25	Up
3.6.1.8	ATP diphosphatase	CCM_07752	52.93	82.38	−0.64	–
4.3.2.2	Adenylosuccinate lyase	CCM_05789	339.7	157.22	1.11	Up
6.3.4.4	Adenylosuccinate synthase	CCM_06768	72.39	17.13	2.08	Up
		CCM_07353	5.3	94.77	−4.16	Down

### Semi-quantitative RT-PCR and Quantitative RT-PCR

2 µg total RNA extracted from each sample was used for cDNA synthesis by ImProm-II™ Reverse Transcriptase (Promega).

The specific primers of each selected gene for semi-quantitative RT-PCR were listed in Table S1. PCR amplification was conducted in a 20 µl reaction system using the following cycling conditions: 95°C for 5 min, followed by 30 cycles of amplification (95°C for 30 sec, 55°C for 30 sec and 72°C for 30 sec), and 72°C for 5 min. The PCR products were identified on 2% agarose gels.

The specific primers of genes for quantitative RT-PCR were listed in Table S2. cDNA amplication was performed in a 20 µl reaction system containing 10 µl Thunderbird qPCR Mix (Toyobo, Osaka, Japan). The cycling conditions were 95°C for 4 min followed by 45 cycles of 95°C for 10 s, 57°C for 15 s and 72°C for 20 s. Relative gene expression levels were analyzed by the 2^−△△CT^ method.

*Actin* gene (CCM_03787) of *C. militaris* was used as the reference gene. Three replicates were performed independently for each gene tested in semi-quantitative RT-PCR and quantitative RT-PCR reactions.

### Protein Extraction

Fresh mycelium and fruiting body (1 g) of *C. militraris* were harvested and ground thoroughly to a fine powder in liquid nitrogen with a ceramic mortar and pestle, respectively. Protein extraction was conducted using trichloroacetic acid/acetone (TCA/acetone) method as previously described [50]. 3.2 mL cold TCA/acetone buffer (10% TCA (w/v) and 0.07% mercaptoethanol in acetone) was added to each power (400 mg), and then the mixture was mixed by inversion. Proteins were precipitated for 1 h at −20°C and pellet was obtained by centrifugation at 15,000 rpm for 15 min at 4°C. The pellet was washed three times with pre-chilled buffer (0.07% 2-ME, 2 mM EDTA, and EDTA-free proteinase inhibitor cocktail tablets (Roche) in 100% acetone) followed by removing all the acetone. The pellet was dried and solubilized in homogenization buffer (0.2 M Tris-HCl buffer, pH 7.8, containing 5 mM EDTA•2Na, 14 mM 2-ME, 10% (v/v) glycerol, and 2 EDTA-free proteinase inhibitor tablets (Roche) per 100 mL of buffer solution in MQ H_2_O). SDS-sample buffer (2.5×, 62 mM Tris (pH 6.8) containing 10% (v/v) glycerol, 2.5% (w/v) SDS, and 5% (v/v) 2-ME, pH 6.8) was added to the mixture to better solublize the proteins. After vortexing and sonication, the supernatant was collected by centriguation at 15,000 rpm for 15 min at 4°C. The supernatant was used for protein quantification by Pierce BCA method.

### One-dimensional Gel Electrophoresis (1-DGE) and Tandem Mass Spectrometry (MS/MS)

150 µg protein of each sample was resolved on a 15% SDS-PAGE after adding bromophenol blue to the protein samples and boiled for 5 min at 95°C. The gels were stained with Coomassie brilliant blue (CBB) R-250 and separated proteins were visualized. Each gel was cut into 5 sections (M_1–5, F_1–5). Gel pieces were washed two cycles by distilled water, then twice in 50 mM NH_4_HCO_3_/50% acetonitrile (ACN) followed by washing in ACN. After the final washing, gel fragments were vacuum-dried in a Speed Vacuum for 10 min. Dried gels were incubated with 10 mM DTT/25 mM NH_4_HCO_3_ at 56°C for 1 h and alkylated with 55 mM iodoacetamide/25 mM NH_4_HCO_3_ at room temperature in the dark for 45 min. After washing with NH_4_HCO_3_, NH_4_HCO_3_/50% ACN and ACN in turn, the gel parts were vacuum-dried again for 10 min. 25 mM NH_4_HCO_3_ containing 0.1 µg/µl trysin was added to the gel tubes and incubated in ice for 30 min. In-gel tryptic degradation was performance with the same digestion buffer without enzyme overnight at 37°C. The reactions were terminated by acidification with 1% TFA to a 0.1% final concentration, then the tubes were centrifuged, the supernatants were collected prior to LC-MS/MS analysis.

Nanoflow reverse-phase liquid chromatography tandem MS was performed using an Easy-nLC system (Bruker, Germany) interfaced directly with the amaZon ETD mass spectrometer via the nanoESI spray source (Bruker, Germany) in the positive ion mode. After sample loading and desalting at 10 µl/min, the peptides were eluted using a linear gradient of solvent B (0.1% formic acid in ACN) in solvent A (0.1% formic acid in H_2_O) from 10% to 35% in 75 min, 35% to 60% in 5 min, and 60% to 90% at 1 min at 400 nl/min, then held at 90% solvent B for an additional 10 min. The ESI-MS was operated in a data-dependent MS/MS mode in which each full MS scan was followed by five MS/MS scans. The nanospray voltage was 1500 V and MS survey scans from m/z 300 to 1,500 were collected in profile mode.

**Figure 6:**
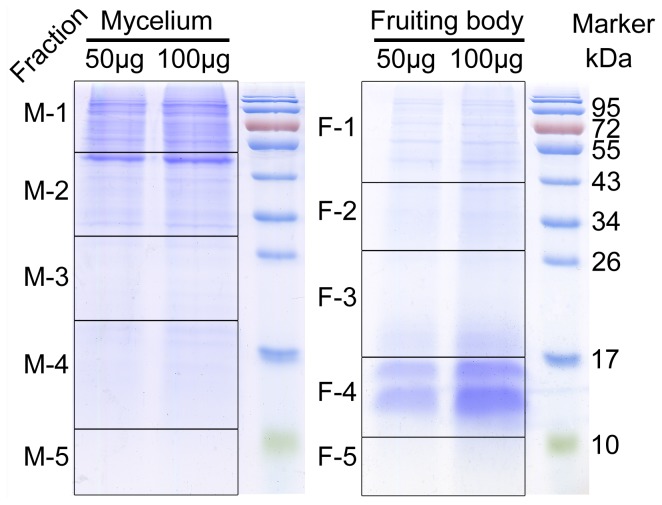
One-dimensional SDS-PAGE separation of *C. militaris* mycelium and fruiting body proteins. The grids indicate how the gel bands (M_1–5, F_1–5) were cut for mass spectrometry. The right of the figure indicates the molecular weight of the markers (kDa).

**Figure 7:**
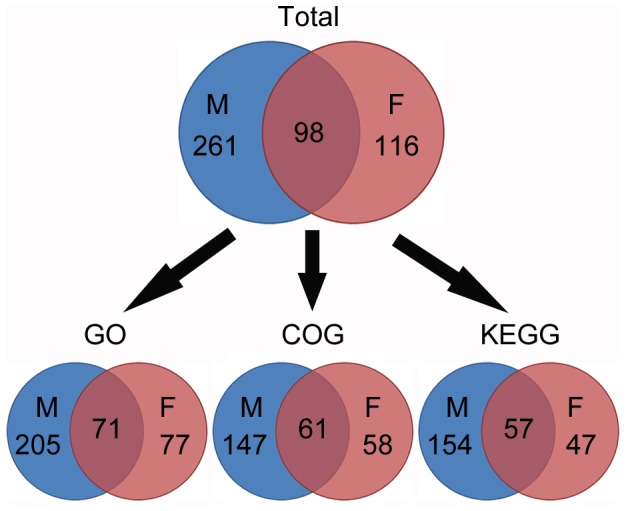
Overview of the protein identified from different samples and their functional annotation summary. M: mycelium; F: fruiting body. The top venn diagram showing the specific and common proteins in mycelium and fruiting body detected by LC-MS/MS. The bottom venn diagrams showing the number of proteins from different samples annotated by GO, COG, and KEGG database respectively.

### Protein Identification

Raw MS data were processed using Flex Analysis (Bruker Daltronics) with the recommended parameters. Proteins identification was performed by searching against the protein database from the sequenced *C. militaris* (http://www.ncbi.nlm.nih.gov/bioproject/41129) using Mascot program (http://www.matrixscience.com) with the initial searching parameters: up to one missed cleavage; no restriction on protein mass; carbamidomethylation as fixed modification, and oxidation of methionine and Pyro-glu from glutamine at N-terminal as variable modification; peptide mass tolerance of ±0.6 Da and fragment mass tolerance of ±1 Da. For individual data analysis, the significance threshold p<0.05 and the Mascot score ≥27 were designed as standards to assign a positive match to a protein in the database.

### Data Deposition

The raw Illumina sequencing data of *C. militaris* was submitted to NCBI Sequence Read Archive (SRA) with the accession number: SRA055440.

**Figure 8:**
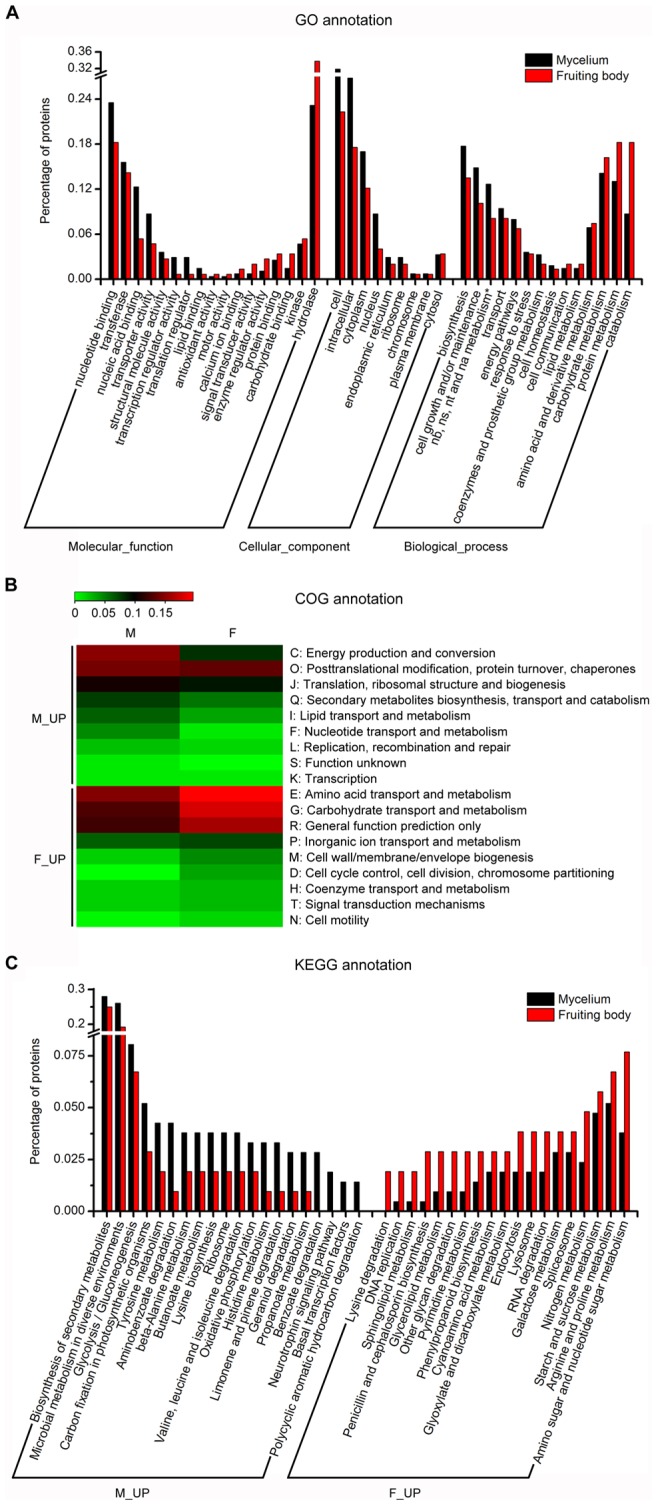
Functional annotation analysis of proteins from *C. militaris* mycelium and fruiting body. (A) Distributions of GO terms of proteins in different samples. The percentage of proteins contained in a particular GO term is shown. * represents nucleobase, nucleoside, nucleotide and nucleic acid metabolism. (B) The heat map of proteins at different stages annotated by COG. The percentage of proteins in each COG category was used to draft the map. (C) Top 39 different KEGG pathway distributions of proteins between mycelium and fruiting body. The y-axis indicates the percentage of proteins involved in a particular KEGG pathway. M_up represents the percentage of proteins in a COG category (B) or KEGG pathway (C) from mycelium is higher than that in fruiting body, F_up is reverse.

**Table 4:** Comparison of the amount of noteworthy proteins in different stages of *C. militaris*.

Kinds of protein	Mycelium	Fruiting body
Proteins involved in cordycepin metabolism	5	3
Lectin	1	2
Superoxide dismutase	2	2
Glycoside hydrolase	12	13

## Results and Discussion

### RNA Sequencing and Mapping

In order to better understand the transcriptomes of *C. militaris* growing in different substrates and the gene expression at different developmental stages, high-throughput Illumina sequencing was performed for RNA samples extracted from *C. militaris* mycelium and fruiting body. The two stages are illustrated in Figure 1A. The pipeline of transcriptome analysis is shown in Figure 1B. After quality filtering, we obtained 6,767,008 and 6,855,896 clean reads of 90 bp in length from mycelium and fruiting body, respectively. About 89.28% of mycelium reads could be mapped to the *C. militaris* genome [30] and 52.53% mapped to gene, while 83.29% of fruiting body reads to the genome and 44.48% to gene (Table 1). Most of the mapped reads got the perfect match (70.57% and 42.03% of mycelium reads to genome and gene respectively, 65.92% and 35.06% of fruiting body reads). Almost all reads were mapped to unique positions except a very small percentage (0.00% to 0.05%).

To evaluate the quality of the transcriptomes, sequencing randomness assessment was performed to detect the random distribution of reads in reference genes. The distribution of reads from two samples was homogeneous in *C. militaris* genes, only the 5′ end of *C. militaris* genes contained relatively lower reads from fruiting body (Figure S1), which indicated the good quality of our sequencing data.

The distributions of genes’ coverage were shown by pie charts in Figure 2. Reads from mycelium and fruiting body transcriptome data sets mapped to 94.88% (9188 of 9684) and 93.04% (9010 of 9684) of *C. militaris* genes, respectively. A large percentage of genes (58% in mycelium, 43% in fruiting body) showed perfect coverage (90% to 100%). Most of genes’ coverage (90.35% in mycelium, 85.13% in fruiting body) is higher than 50%. Overall, high quality sequencing and mapping results were obtained.

### Alternative Splicing and Novel Transcripts Prediction

Alternative splicing (AS) is essential for protein diversity and functional complexity [64], [65]. We examined four basic AS events of each sample, including exon skipping (ES), intron retention (IR), alternative 5′ splicing (A5SS) and alternative 3′ splicing (A3SS). 654 AS events in mycelium and 424 in fruiting body were identified (Figure 3A). Like other fungi, the IR is the major class which accounts for about 55% (366 in mycelium, 234 in fruiting body) of all AS events in *C. militaris*, but still lower than that in *Aspergillus oryzae* (91.56%) [66] and *Tuber melanosporum* (94%) [42]. Because some genes produced two or more AS events, totally 738 (7.6%) *C. miltaris* genes (552 in mycelium, 368 in fruiting body) were estimated to undergo AS (Figure 3B). This percentage is lower in *Ustilago maydis* (3.6%) [67] and *Cryptococcus neoformans* (4.2%) [68], but higher in *A. oryzae* (8.55%) [66] and *T. melanosporum* (15.4%) [42]. When AS events were compared between different developmental stages of *C. militaris*, we found the number of genes involved in AS in mycelium was higher than that in fruiting body. Particularly, 556 genes (370 in mycelium, 186 in fruiting body) were stage-specific, suggesting that AS is developmentally regulated (Figure 3B, Table S3). Several mycelium-specific AS genes encoded protein kinase, mitochondrial proteins and fungal transcriptional regulatory proteins. And a hydrophobin gene (CCM_03537) was identified in fruiting body-specific AS genes. It has been reported that hydrophobin genes play important roles in the fruiting body formation of fungi [69], [70]. These results suggested that stage-specific AS genes might have important functions in fungi development.

In addition, novel transcripts can be determined by high-throughput sequencing to enrich the present database [62]. We predicted 3318 and 2932 novel transcripts in mycelium and fruiting bodying, respectively, of which about 20% (734 in mycelium, 576 in fruiting body) were longer than 500 bp (Figure 3C, Table S4).

### Analysis of Differentially Expressed Genes (DEGs)

Mycelium and fruiting body are two major developmental stages of *C. militaris*. RNA-seq data and RPKM methods (see methods) were used to identify genes showing significant changes in expression during the two stages. We found 2712 genes were differentially expressed between mycelium and fruiting body, including 2113 and 599 genes up-regulated in mycelium and fruiting body respectively (Figure 4A and B, Table S5). This overall distribution was consistent with the previous study in mycelium culturing on Sabouraud dextrose broth (SDB) and fruiting body culturing on caterpillar pupae [30], but the detailed expression condition was different. Among the 100 most highly expressed genes, although still more than 10% pathogen-host interaction genes (11 in mycelium, 20 in fruiting body) could be found, indicating the main characteristic of *C. militaris* remained albeit the culturing substrate was changed, more hypothetical proteins and glycoside hydrolases (GH) genes were seen in our data (Table S6). Glycoside hyrolases that cleave oligo- and polysaccharides have been reported to be important in biology of fungi like *Tuber melanosporum* [43], *Trichoderma reesei* [38] and *Postia placenta* [45]. Further study could be targeted on these GH and unknown function genes.

To validate the gene expression profile, 17 genes were randomly selected to perform semi-quantitative RT-PCR (Figure 4C, Table 2). CCM_03787 was the *actin* gene in *C. militaris* and was used as a reference. Except the CCM_07787 (Mannose-binding lectin), the PCR results of 16 genes were in accordance with the transcriptome data, including 3 genes commonly expressed in two stages, 6 genes up-regulated in mycelium and 7 genes up-regulated in fruiting body. This indicated that the transcriptome data and the DEGs analysis are reliable.

The GO function enrichment analysis globally provided the function terms which significantly enrich in DEGs comparing to the genome background. All DEGs were mapped to the GO terms in three main categories (biological process, cellular component and molecular function) in the GO database, ten terms that show the smallest p value were displayed in Figure 4D. Adenyl nucleotide binding, ATP binding and catalytic activity in molecular function and integral to membrane in cellular component were significantly enriched in DEGs (p≤0.05), suggesting the importance of these terms in different developmental stages. Besides that, different genes usually cooperate to exercise their biological functions. Pathway-based analysis helps us to further understand biological functions of genes. KEGG pathway enrichment analysis was carried out to identify significantly enriched metabolic pathways or signal transduction pathways in DEGs. Ten pathways that showed the smallest Q value were selected, in which starch and sucrose metabolism, amino sugar and nucleotide sugar metabolism showed significant enrichment (Q ≤0.05) (Figure 4E). These metabolism pathways associated with energy production were indispensable for fungi growth. Comparing the GO annotation of DEGs between mycelium and fruiting body indicated that the annotation percentages of hydrolase, nucleotide binding, nucleic acid binding, transferase, kinase, transcription regulator activity, intracellular, nucleus, nucleobase, nucleoside, nucleotide and nucleic acid metabolism, protein metabolism, cell growth and/or maintenance in mycelium up-regulated genes were higher than that in fruiting body up-regulated genes, while terms involved in transporter activity, signal transducer activity, carbohydrate binding, nutrient reservoir activity, cytoplasm, external encapsulating structure, biosynthesis, carbohydrate metabolism, lipid metabolism, amino acid and derivative metabolism, coenzymes and prosthetic group metabolism, cell communication showed higher levels in fruiting body (Figure 4F). This analysis suggested that intracellular nucleotide binding and metabolism, transcription regulator activity were more active in mycelium, which might be a preparation for the later fruiting process. Signal transduction, carbohydrate and lipid metabolism were more important for *C. militaris* fruiting body growth, because more energy and nutrient were needed in this process and exactly the rich carbohydrate in the rice medium could be well utilized. In addition, the external structure may be necessary for *C. militaris* to maintain its insect pathogenicity. Previous study demonstrated that the Zn2Cys6 type TFs was involved in the switch of fruiting structures production of *C. militaris* culturing on pupae [30]. The expression difference of transduction and transcription factors between mycelium on PDA and fruiting body on rice was analyzed in our research. Interestingly, we found that CCM_06752 (Alpha-pheromone receptor PreB) and CCM_01809 (Zn2Cys6-type TF) were up-regulated in fruiting body, indicating their importance for fruiting body growth (Table S7).

### Putative Cordycepin Metabolism Pathway

Cordycepin (3′-deoxyadenosine), one of the main bioactive compounds of *C. militaris*, has been reported to have a variety of medicinal value [19]–[24]. The *C. militaris* is scarce in nature and expensive in the market. The production of cordycepin from *C. militaris* fruiting body cannot reach commercial needs, thus many researches were concentrated on obtaining cordycepin from cultured mycelium of *C. militaris* and now more advanced approaches have been employed in how to improve its productivity [71]–[76]. Hopefully, the active components of cultured *C. militaris* have been determined to be similar as the natural ones including cordycepin [27]–[29].

Both adenine and adenosine could be the precursor of cordycepin, and the addition of them to the culture medium of *C. militaris* could increase the productivity of cordycepin [75]. Thus, we extracted the adenine metabolism pathway from the purine metabolism pathway using KEGG information and took the previously associated forecast by other researchers into account [30]. The putative cordycepin metabolism pathway was shown in Figure 5A. To further understand this pathway and give information for higher yield of cordycepin, the expression difference of genes involved in this pathway between mycelium cultured on PDA and fruiting body cultured on rice were studied (Figure 5A, Table 3). Most of the enzymes involved in the pathway were up expressed in mycelium such as ribonucleotide reductase, adenosine kinase, pyruvate kinase, 5′-nucleotidase, purine nucleosidase, adenine deaminase, AMP deaminase, and adenylosuccinate lyase. Only a purine nucleoside phosphorylase (CCM_04505) and an adenylosuccinate synthase (CCM_07353) were up expressed in fruiting body. To validate the data, 22 genes in the pathway were randomly selected to perform qRT-PCR. The results showed 18 genes were in accordance with the transcriptome data except CCM_05761, CCM_02831, CCM_06768 and CCM_07353 (Figure 5B). CCM_05761 and CCM_02831 were up expressed in fruiting body while CCM_07353 was only detected in mycelium (Not shown in the figure). CCM_06768 showed no significant difference between mycelium and fruiting body. In addition, inosine monophosphate (IMP) synthesis pathway, which was the upstream of the putative cordycepin metabolism pathway, was analyzed (Figure 5A). The enzymes involved in four steps were up-regulated in mycelium while only one step was up-regulated in fruiting body. Some of the intermediates in this pathway were functionally related to known major pathways. For example, phosphoribosyl pyrophosphate (PRPP) was related to pentose phosphate pathway, ribosylamine-5P was related to alanine, aspartate and glutamate metabolism pathway, 5-aminoimidazole ribotide (AIR) was related to thiamine metabolism pathway and 5-aminoimidazole-4-carboxamide ribotide was related to histidine metabolism pathway. These pathways were fundamental and important for fungi growth and development. Together, cordycepin metabolism pathway seemed to be much active in mycelium of *C. militaris* and could be associated to many familiar pathways. Thus, production of the cordycepin from the mycelium of *C. militaris* in a large scale is promising and optimization of the program is needed.

### Proteomics Characterization

In order to deeply understand the difference between mycelium and fruiting body of *C. militaris*, proteomes of the two stages were also analyzed. The pipeline of proteome analysis was shown in Figure 1B. Total proteins of mycelium and fruiting body were separated on 15% SDS-PAGE respectively followed by cutting each gel into five sections (Figure 6). In-gel trypsin-digested peptides were performed nESI-LC-MS/MS. The proteins were then identified by searching against *C. militaris* genome database using Mascot program. 359 and 214 non-redundant proteins were detected from mycelium and fruiting body respectively, among which 98 proteins were found in both samples including glycoside hydrolase (CCM_07342), heat shock protein 70 (CCM_05489), enolase (CCM_06659), glucose-6-phosphate isomerase (CCM_09292), tansketolase (CCM_00345) (Figure 7 and Table S8). Glycoside hydrolases catalyze the hydrolysis of the glycosidic linkage to release smaller sugars and play significant role in biomass degradation by some fungi [38]. Heat shock proteins have been reported to protect the cells against stress conditions and be potentially involved in host-pathogen interaction [77]. Enolase, glucose-6-phosphate isomerase and tansketolase, associated with sugar metabolism, are important in both developmental processes.

### Functional Annotation Analysis of Proteome Difference

Functional annotations were performed to analyze the protein expression difference between mycelium and fruiting body. 276, 208, 211 proteins from mycelium and 148, 119, 104 proteins from fruiting body were annotated by GO, COG and KEGG database respectively (Figure 7). GO categories comparison indicated that the percentages of proteins involved in nucleotide binding, transferase, nucleic acid binding, transporter activity, transcription regulator activity, translation regulator, cell, intracellular, cytoplasm, nucleus, biosynthesis, cell growth and/or maintenance, nucleobase, nucleoside, nucleotide and nucleic acid metabolism, transport, energy pathways were higher in mycelium than in fruiting body. However, the terms of hydrolase, kinase, carbohydrate binding, protein binding, enzyme regulator activity, signal transducer activity, catabolism, protein metabolism, carbohydrate metabolism, amino acid and derivative metabolism, lipid metabolism, cell communication showed opposite result (Figure 8A). Together with the GO annotation of the transcriptomes (Figure 4F), we demonstrated that intracellular nucleotide binding and metabolism, transcription regulator activity, cell growth and maintenance, transport were more active in mycelium while carbohydrate binding and metabolism, lipid metabolism, signal transduction were more active in fruiting body at both transcriptional and translational level, which was consistent with the need for each developmental stage.

The detected proteins were classified into 18 clusters of orthologous groups (COG) categories. The heat map showed the classification difference between the two proteomics intuitively (Figure 8B). The percentages of proteins involved in the categories of Energy production and conversion, Posttranslational modification, Protein turnover, Chaperones, Translation, Ribosomal structure and biogenesis, Nucleotide transport and metabolism were higher in mycelium while the categories of Amino acid transport and metabolism, Carbohydrate transport and metabolism, Cell wall/membrane/envelope biogenesis, Signal transduction mechanisms showed higher level in fruiting body proteome. The overall functional bias was similar with the GO analysis.

KEGG pathway analysis was performed to further study the pathway difference between the proteome of different developmental stages. 39 pathways with largest difference were shown in Figure 8C. Compared to fruiting body, the pathway of Biosynthesis of secondary metabolites, Microbial metabolism in diverse environments, Glycolysis/Gluconeogenesis, Tyrosine, histidine and beta-alanine metabolism, Basal transcription factors, Small molecular compounds degradation including aminobenzoate, benzoate, geraniol, limonene and pinene, Oxidative phosphorylation were more active in mycelium. But the pathways of Amino sugar and nucleotide sugar metabolism, Starch and sucrose metabolism, Spliceosome, Glyoxylate and dicarboxylate metabolism, RNA degradation and Other glycan degradation were enriched in fruiting body. The result suggested that the pathways involved in biosynthesis, transcription and translation were more important in mycelium stages while carbohydrate metabolism and some degradation pathways were conducive to fruiting body growth.

To further analyze the proteome data, we investigated some noteworthy proteins in *C. militaris* mycelium and fruiting body. Here, we found 5 proteins involved in cordycepin metabolism in mycelium proteome and 3 in fruiting body (Table 4), which was consistent with our speculation from the transcriptomes that cordycepin metabolism was more active in mycelium than fruiting body (Figure 5). Moreover, mushroom lectins have been studied for several years, and many of them possess antitumor and immunomodulatory activities [78]–[80]. A lectin purified from *C. militaris* exhibits mitogenic activity against mouse splenocytes [16] and a heamagglutinin displays antitumor and anti-HIV-1 effects [81]. Superoxide dismutase, an antioxidant enzyme, has powerful anti-inflammatoy and pharmacological activities [82], [83]. A Cu, Zn superoxide dismutase gene from *C. militaris* had been cloned and expressed [84]. In our proteomic research, though fewer total proteins were detected in fruiting body compared with mycelium, more lectins and the same amount of superoxide dismutase were identified, which may contribute to the medical value of the *C. militaris* fruiting body (Table 4). Moreover, 12 glycoside hydrolases were detected in mycelium proteome and 13 in fruiting body, demonstrating their importance in *C. militaris* growth, especially in fruiting body stage. These results will support the exploitation of the effective protein components in *C. militaris*.

### Conclusions

This study reported and analyzed the difference between mycelium and fruiting body of artificial cultivated *C. militaris* by transcriptome and proteome analysis. Functional annotations revealed that intracellular nucleotide binding and metabolism, transcriptional regulation and translation were more active in mycelium while carbohydrate metabolism and signal transduction were more active in fruiting body. Fruiting body growth needs more energy and the rice medium can support rich carbohydrate. Besides, putative cordycepin metabolism pathway may be more active in mycelium, which is correlated with cordycepin production. Alternative splicing and novel transcripts prediction also enriched the database of *C. militaris*. Noteworthy proteins such as lectin and superoxide dismutase were detected to offer clues for further study of medicinal ingredients. In summary, our research will promote the developmental and pharmacological research of *C. militaris*.

## Supporting Information

Figure S1 **Reads distribution of two sequenced samples in** ***C. militaris*** **genes.** (A) Mycelium. (B) Fruiting body. The x-axis indicated the relative position of sequenced reads in the *C. militaris* genes. The orientation of genes: 5′ end to 3′ end.
(TIF)

Table S1 **Primers used for semi-quantitative RT-PCR.**
(DOC)

Table S2 **Primers used for quantitative RT-PCR.**
(DOC)

Table S3 **Genes with developmental stage-specific alternative splicing.** ES, exon skipping; IR, intron retention; A5SS, alternative 5′ splice site; A3SS, alternative 3′ splice site.
(XLS)

Table S4 **List of novel transcripts identified from mycelium and fruiting body by RNA-Seq.**
(XLS)

Table S5 **Information of differentially expressed genes (DEGs) between mycelium and fruiting body of** ***C. militaris*** **.** FDR ≤0.001 and |log_2_RPKM ratio| ≥1 were set as the threshold. 2712 DEGs were determined in which 2113 up-regulated in mycelium and 599 in fruiting body.
(XLS)

Table S6 **The 100 top expressed genes in different developmental stages.** PHI genes, pathogen-host interaction genes predicted by blastp against the PHI database (http://www.phi-base.org/) with a cut-off *E* value of 1e-5. GH genes, glycoside hydrolases genes predicted by blastp against CAZy database (http://www.cazy.org/) with a cut-off *E* value of 1e-20 and identity higher than 50%.
(XLS)

Table S7 **Comparison of the expression levels of genes putatively involved in signal transduction and transcription controls in** ***C. militaris.*** No significant expression difference between mycelium and fruiting body.
(XLS)

Table S8 **Detailed information of the proteins from mycelium and fruiting body identified by nESI-LC-MS/MS.** Asterisks indicate the commonly identified proteins in mycelium and fruiting body of *C. militaris*.
(XLS)
