# Supplementary figures and images for: Genome-Wide Transcriptome and Proteome Analysis on Different Developmental Stages of Cordyceps militaris
Source: PLoS One. 2012 Dec 14;7(12):e51853. doi: 10.1371/journal.pone.0051853 (PMC3522581; doi:10.1371/journal.pone.0051853)

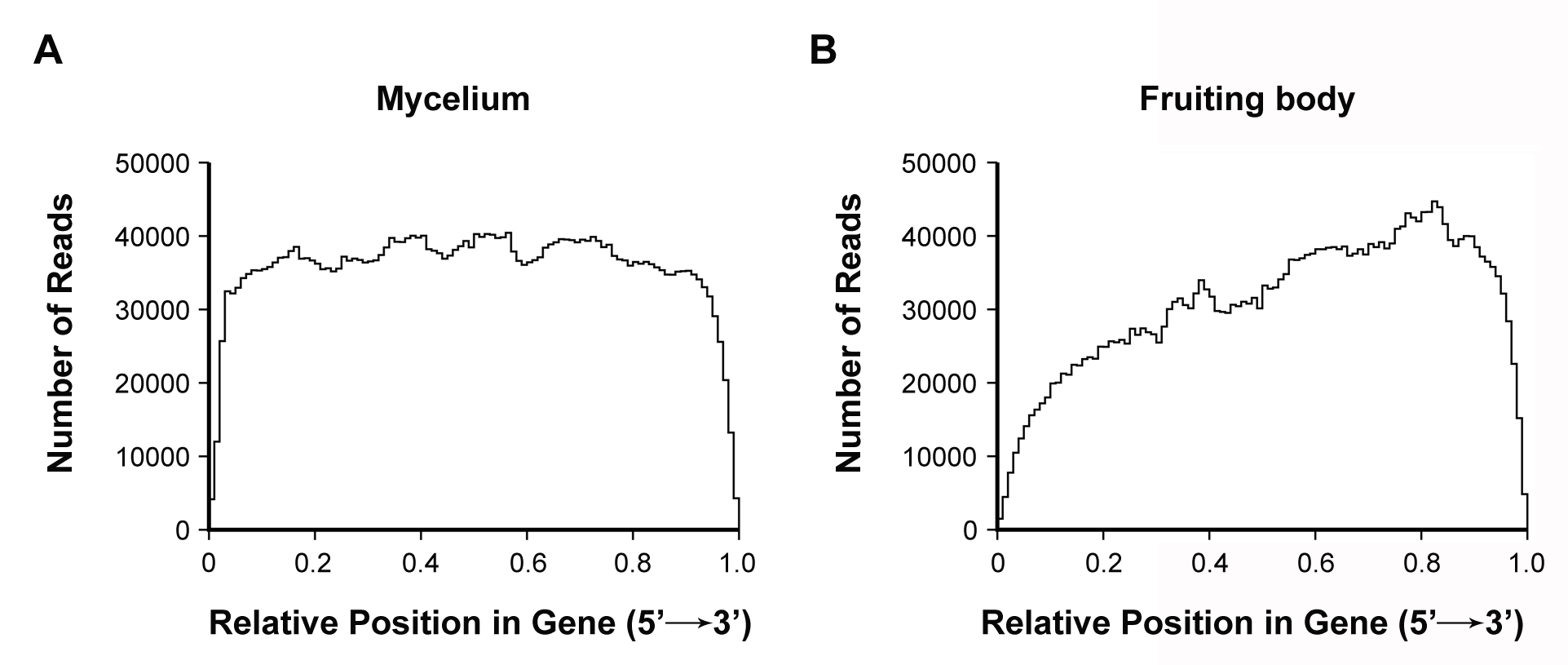

Supplement: Figure S1 — Reads distribution of two sequenced samples in C. militaris genes. (A) Mycelium. (B) Fruiting body. The x-axis indicated the relative position of sequenced reads in the C. militaris genes. The orientation of genes: 5′ end to 3′ end. (TIF) [file pone.0051853.s001.tif]
