# Supplementary material for: Genome-Wide Transcriptome and Proteome Analysis on Different Developmental Stages of Cordyceps militaris
Source: PLoS One. 2012 Dec 14;7(12):e51853. doi: 10.1371/journal.pone.0051853 (PMC3522581; doi:10.1371/journal.pone.0051853)
Supplement: Table S1 — Primers used for semi-quantitative RT-PCR. (DOC) [file pone.0051853.s002.doc]

**Table S1** Primers used for semi-quantitative RT-PCR.

| **Gene** | **Primers (F, forward; R, reverse)** |
| --- | --- |
| CCM_03787 | F: GCGTGAAATCGTTCGTGAC |
|  | R: GGCGAGACCAGGGTACATAGTG |
| CCM_01180 | F: TCTCATCAATTACCGCAAAGG |
|  | R: TATCACATCGTCGCAGTTCTC |
| CCM_00072 | F: CGCCAAGATTGTTGAGGAG |
|  | R: GCGTTTCGGGTAGGGTATG |
| CCM_01779 | F: AAGAACGAGGACGAGTGGCT |
|  | R: CGGCGATGAAGAAGAATATGAC |
| CCM_00622 | F: GGTCGTCTTCTTGCTGGTG |
|  | R: TTGGCGTTATCGTCTGAGG |
| CCM_01829 | F: CATTACCGACATCATCACC |
|  | R: ACAGGCACAGCCACATC |
| CCM_08261 | F: CATTCTGAAGGAACTCAACCCT |
|  | R: ATACTGCGGCATTGTCTCGG |
| CCM_02883 | F: TTTCCCAAGGTCCATCCAAC |
|  | R: CATCAATCAAGCCAGCGACT |
| CCM_07787 | F: CAAACCAGCGGAGACCAG |
|  | R: GAGCCAAGTGAGAAGAGCG |
| CCM_01530 | F: GCGGTACATTCCTGGCTGGTT |
|  | R: GCTCGGTCCTGAAAGGTTGG |
| CCM_05618 | F: GATCAAATGCGATCCCGACT |
|  | R: GCCAGGCACAACATAAGACC |
| CCM_09501 | F: GACGCACATCAGCGACTACCAC |
|  | R: CGCCCTGAATCACCTCCACAT |
| CCM_02051 | F: CGTGGTGCCCAACTTTCC |
|  | R: GCCTATTCTCGCCTCTTTCG |
| CCM_07839 | F: CGACCAAGTCTGGCGATG |
|  | R: CGAACTGGCCGGTACGAAT |
| CCM_08095 | F: CCAAAGCCACCTTCTTCATC |
|  | R: GCGTCTCCACGGTCTCATAG |
| CCM_00274 | F: CACGACATTGGCGAGGAC |
|  | R: GTTGGACACGGCGTTGAT |
| CCM_03537 | F: TCCTCTACTCCAACCCCATCTG |
|  | R: ATAATGGGAAGAACGCAGCACC |
| CCM_05117 | F: TTCGCCAAGTACAAGAACACTCT |
|  | R: TTGCTCATCAAAGCCTTAACCT |
