# Supplementary material for: Genome-Wide Transcriptome and Proteome Analysis on Different Developmental Stages of Cordyceps militaris
Source: PLoS One. 2012 Dec 14;7(12):e51853. doi: 10.1371/journal.pone.0051853 (PMC3522581; doi:10.1371/journal.pone.0051853)
Supplement: Table S2 — Primers used for quantitative RT-PCR. (DOC) [file pone.0051853.s003.doc]

**Table S2** Primers used for quantitative RT-PCR.

| **Gene** | **Primers (F, forward; R, reverse)** |
| --- | --- |
| CCM_03787 | F: GTCCACCTTCCAGCAAATGT |
|  | R: GGTGTGGTGCCAAATCTTCT |
| CCM_05761 | F: GCTGCTTCTACGGTTTCCAG |
|  | R: GGGCAAAGGTAGACTGCTTG |
| CCM_01863 | F: CTATGGAGCTTGCCAAGGAG |
|  | R: TAAGGCTCGAAGCATTCGTT |
| CCM_04505 | F: ACATCAACATCGCCTTCCTC |
|  | R: TCCATCGGCTTGATATCCTC |
| CCM_00088 | F: CCCGACGTCATTGTAGGTCT |
|  | R: TCCTTGCCGTACTCCTTGAC |
| CCM_02716 | F: CTCACATCACCGACACCAAC |
|  | R: TTGGCCTTGAGCTCGTACTT |
| CCM_07110 | F: CCAACTCGCCGTCTGATATT |
|  | R: TGGCACAAATTCTTGAGCAG |
| CCM_02335 | F: ACCTGATCCGCGACTACATC |
|  | R: AACTTGTACGCCTGGTCGAG |
| CCM_01353 | F: TCGAAGAATTCCAGGACCAG |
|  | R: TCATCAGTGCTTTGGTCTCG |
| CCM_07126 | F: AGAACGCCCAGAAGGAGATT |
|  | R: AGTCTCGAGATGCTCCTTGG |
| CCM_02831 | F: ATCTGGGAGATTCGCAACAC |
|  | R: TCCTTGCCAGACTTGTCCTT |
| CCM_00622 | F: GTCAGTGGCCGAGTTCTCTC |
|  | R: GCGCGGATGAAACAATATCT |
| CCM_04644 | F: CCGACTTCAAGAACCTCAGC |
|  | R: CCAATGTTGGACTCCTTGGT |
| CCM_06141 | F: GTCACATACGGCAACGTGTC |
|  | R: TTGAGGTCCGTCTCTTCCAG |
| CCM_09682 | F: GCTCCCTGACCAATGTCG |
|  | R: GGTCGATGACGATGTTGAACT |
| CCM_07169 | F: GACATGGACCACTGCATCAC |
|  | R: GCGTCAAAGAGGTTCTCGTC |
| CCM_02911 | F: GCGGAGCAACCAACTATACC |
|  | R: GGCCACTTCTGCTTGAACTC |
| CCM_09680 | F: TGGCCGAAGTCTTTGCTAGT |
|  | R: CCACTCGGCCATTTGATACT |
| CCM_00441 | F: TGCTGATGACCTGAAACTCG |
|  | R: GAATTTCGTGGACGTTGGTT |
| CCM_07752 | F: AGCCAGTTACGACACCCATC |
|  | R: GTCGTCGGTTTCGGTAGTGT |
| CCM_05789 | F: TAGGGAATGGAAGGCAACAC |
|  | R: CGAAGCTTGAGTTCCCGTAG |
| CCM_06768 | F: ATGCCGTCACCTTTATGGAG |
|  | R: GACAACGCCAATGATTTCCT |
| CCM_07353 | F: CCTCATCACCACATCACCAG |
|  | R: GAAAAGCACCAGACGAGGAG |
